# MicroRNA Regulation and Tissue-Specific Protein Interaction Network

**DOI:** 10.1371/journal.pone.0025394

**Published:** 2011-09-27

**Authors:** Wenliang Zhu, Lei Yang, Zhimin Du

**Affiliations:** 1 Institute of Clinical Pharmacology, the Second Affiliated Hospital of Harbin Medical University, Harbin, China; 2 Department of Orthopedics, the First Affiliated Hospital of Harbin Medical University, Harbin, China; Niels Bohr Institute, Denmark

## Abstract

**Background:**

‘Fine-tuning’ of protein abundance makes microRNAs (miRNAs) pervasively implicated in human biology. Although targeting many mRNAs endows the power of single miRNA to regulate complex biological processes, its functional roles in a particular tissue will be inevitably restricted because only a subset of its target genes is expressed.

**Methods:**

Here, we analyze the characteristics of miRNA regulation upon target genes according to tissue-specific gene expression by constructing tissue-specific protein interaction networks for ten main types of tissues in the human body.

**Results:**

Commonly expressed proteins are under more intensive but lower-cost miRNAs control than proteins with the tissue-specific expression. MiRNAs that target more commonly expressed genes usually regulate more tissue-specific genes. This is consistent with the previous finding that tissue-specific proteins tend to be functionally connected with commonly expressed proteins. But to a particular miRNA such a balance is not invariable among different tissues implying diverse tissue regulation modes executed by miRNAs.

**Conclusion:**

These results suggest miRNAs that interact with more commonly expressed genes can be expected to play important tissue-specific roles.

## Introduction

Implement of complex processes in biology are tremendously dependent on interactions between proteins. Drafting global human protein interaction map, researchers have the opportunity to explore how proteins fulfill their cellular functions [1]–[3]. Although protein interactions are usually mapped into a static protein interaction network (PIN), cautions should be taken that a real PIN is always in dynamic states [4]. Dynamic PIN ensures cell show good robustness when facing various kinds of perturbations from the external environment [5]. An important controllable variable is protein abundance. In human, microRNAs (miRNAs) have emerged as vital regulators in ‘fine-tuning’ of protein abundance and are involved in nearly all biological processes [6].

The incompletely complementary binding mechanism of miRNA-gene interaction enables a single miRNA recognize and target many mRNA genes [7]. In the last several years, the repository of the validated miRNA targets has been exponentially growing due to the important functions of miRNAs continuously brought to light [8]. And furthermore, new target prediction methods have been developed with improved accuracy [9]. These greatly enrich our understanding about the biological functions of miRNAs. Recently, several studies about miRNA regulation of cellular networks have been published in the context of PINs [10]–[12]. The relationship between miRNA regulation and global PIN has been revealed by investigating the influence of miRNAs to the PIN dynamics. Most researchers chose to build PINs in the proteome-wide scale. However, very little is known about the characteristics of miRNA regulation upon PINs with gene expression restriction (or in the tissue-restricted scale). In reality, not all of the genes encoded in human genome can express within a particular tissue [13]. This determines that interactions between proteins encoded by co-expressed genes can occur. Bossi and Lehner recently confirmed that commonly expressed proteins are also essential for tissue-specific biology as tissue-specific proteins tend to be directly connected with them [13].

Owing to only a subset of target genes of a miRNA being expressed in a tissue, limited functional roles of the miRNA can be expected. In the present study, we apply a network biology approach to miRNA regulation in context of tissue-specific PINs in which only proteins of experimentally validated tissue expression are represented as nodes. Defining the tissue specificity of PINs, we are able to reveal the characteristics of condition-dependent miRNA-gene interactions. Surprisingly, we find relatively more mature miRNA regulation upon commonly expressed proteins than those tissue-specific ones. More miRNA-gene interactions are directed to the genes encoding commonly expressed proteins, but less number of different miRNAs per gene used. Tissue-specific proteins tend to be directly connected with commonly expressed proteins. This not only indicates the essential roles of commonly expressed proteins in tissue-specific biology, but also implies the possible balance of miRNA regulation between commonly expressed and tissue-specific proteins. We further uncover that miRNAs regulating more commonly expressed proteins usually also affect the expression of more tissue-specific proteins although in different tissues such a balance varies to a particular miRNA. Our results suggest miRNAs with enriched regulation on the core cellular components comprised by commonly expressed proteins can also be expected to play important tissue-specific roles.

## Results

### Most tissue-specific proteins are located in the periphery and tend to interact with commonly expressed proteins

**Figure 1:**
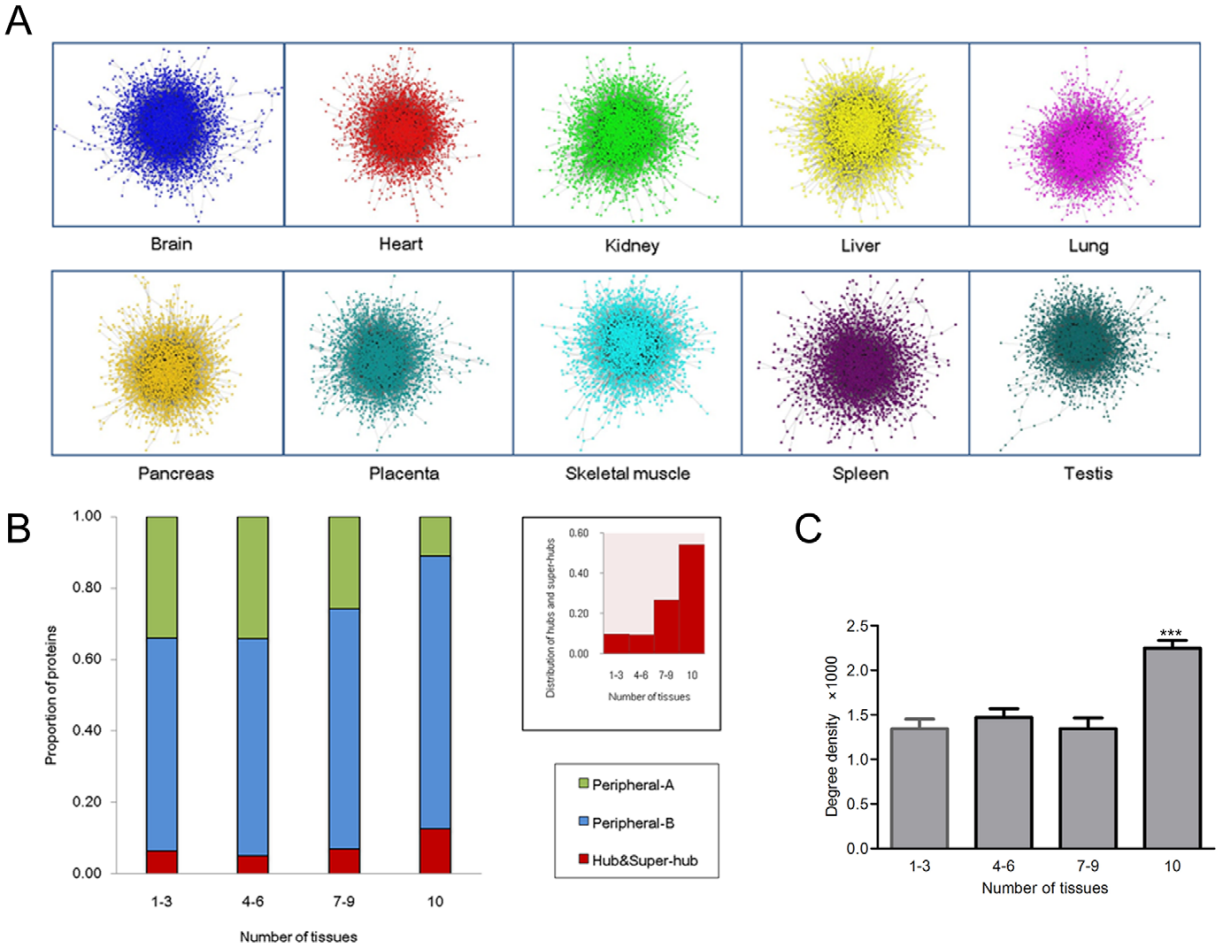
Tissue-specific proteins tend to interact with commonly expressed proteins. A. Tissue-specific protein interaction networks for the 10 main human tissues. B. The relationship between protein expression specificity and protein interaction degree (Peripheral-A: degree  = 1; Peripheral-B: 20> degree >1; Hub: 100> degree ≥20; Super-hub: degree ≥100), right upper: Distribution of hub and super-hub proteins in different protein subunits. C. The degree densities of intra-subunit of tissue-specific proteins and between tissue-specific subunit and other protein subunits (***1-3 versus 10, *p*<0.001).

To generate tissue-specific human PINs, we retrieved the integrated protein interaction data [14] from 6 popular protein interaction databases (BIOGRID, INTACT, MINT, DIP, BIND and HPRD) for the proteins with experimentally validated expression [15] in the 10 main human tissues (Figure 1A). We analyzed the topological feature of the 10 created tissue-specific PINs and distributed proteins represented as nodes into four categories according to their interaction degree (see “Methods”). And furthermore, all proteins were also categorized in the context of tissue expression specificity. Totally 1133 proteins were defined as commonly expressed proteins because they were found expressed in all of the 10 tissues. We found that more than half of the hubs and super-hubs belonged to the commonly expressed subunit (Figure 1B, right upper). And overall, commonly expressed proteins have higher degrees than proteins in the other subunits (Figure 1B). These suggest the core roles of commonly expressed proteins in cellular biological processes. Compared to commonly expressed proteins, tissue-specific proteins that express only in less number of tissues (n≤3) would likely to be located in the periphery of the network due to the lower number of interactions they make (Figure 1B). Our result is consistent with the previous finding [13].

We also evaluated the interaction propensity of intra- and inter-subunits of proteins by introducing the concept of degree density. Considering the maximum possible number of protein interactions, degree density quantitatively describes how two classes of proteins are closely interacted with each other (see “Methods”). Figure 1C shows the degree densities of intra-subunit of tissue-specific proteins and between tissue-specific subunit and other protein subunits. The significant propensity of tissue-specific proteins interacting with commonly expressed proteins was found (*p*<0.001). Tissue-specific proteins are often considered as the direct implementers of tissue-specific biology. However, our results once again confirm that they are not isolated, and commonly expressed proteins are also essential to the tissue-specific biology [13].

### Commonly expressed proteins are under more mature miRNA monitoring than tissue-specific proteins

**Figure 2:**
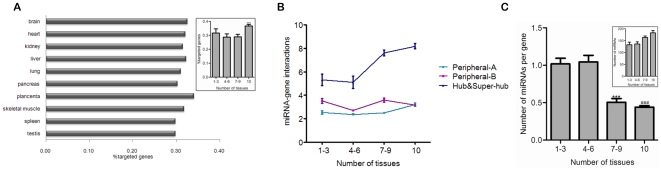
Commonly expressed proteins are under more mature miRNA monitoring than tissue-specific proteins. A. Percentage of genes targeted by at least one miRNA in different tissue-specific PIN, right upper: Distribution of miRNA-gene interactions (%) in different protein subunits (***1–3 versus 10, *p*<0.001). B. The relationship between miRNA-gene interactions in different protein subunits and protein interaction degree. C. The relationship between protein expression specificity and the number of different miRNAs per gene (###1–3 versus 7–9, *p*<0.001 and ***versus 10, *p*<0.001), right upper: Counts of miRNAs in different protein subunits (***1–3 versus 10, *p*<0.001).

We assigned the miRNAs with validated expression in each tissue [16] with their literature reported [8] and high confidently validated [9] target genes. In the result, 14032 interactions were found between 294 miRNAs and 4037 protein-encoding proteins. The miRNA-gene interaction data was imported into the 10 tissue-specific PINs respectively. No significant variation in co-expression of different tissue-specific gene subunits with their corresponding miRNAs was found (Table S1). This result further revealed that gene targets of different tissue specificity might share the same mechanism of miRNA-mediated posttranscriptional regulation [17]. We found that approximately 30% of proteins are under miRNA control in all of the PINs (Figure 2A). Notably, commonly expressed proteins are more favored by miRNAs compared to the other protein subunits (*p*<0.001). It is easy to understand that hub and super-hub proteins are focused upon more miRNA-gene interactions because of their comparatively important biological roles (Figure 2B). Especially, to those universally expressed hub and super-hub proteins largest number of miRNA-gene interactions was found on average. We consider that this strengthened miRNA regulation is in line with their extensive and crucial roles in human biology [18].

Another interesting finding is that the number of different miRNAs per targeted gene encoding commonly expressed protein is quite lower compared to that of tissue-specific protein encoding genes on average (Figure 2C, *p*<0.001) despite commonly expressed proteins are favored by more miRNAs than tissue-specific proteins (Figure 2C, right upper, *p*<0.001). Coupled with the finding showed in Figure 2B, we speculate that this great contrast might indicate more mature miRNA regulation upon commonly expressed proteins, which are believed to be the earlier products of biological evolution than tissue-specific proteins. Mature miRNA regulation is reflected by that although more miRNA-gene interactions per gene are adapted, less number of different miRNAs per gene used. On one hand, intensive miRNA control of protein expression maintains the overall cellular stability; on the other hand, altered expression of a single miRNA enable cells respond appropriately to environmental perturbation with lower cost.

### MiRNAs that regulate more commonly expressed proteins also affect expression of more tissue-specific proteins

**Figure 3:**
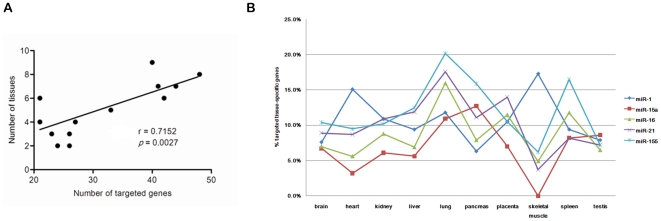
MiRNAs that regulate more commonly expressed proteins also affect expression of more tissue-specific proteins. A. The positive correlation between the number of targeted commonly expressed genes of miRNAs (n>20) and the number of tissues in which a relatively high number of tissue-specific proteins regulated by miRNAs (n≥8) can be found (Pearson correlation coefficient *r* = 0.7152, *p* = 0.0027, n = 15). B. The percentage of tissue-specific proteins regulated by miRNAs with enriched control upon commonly expressed proteins (n≥40) in each tissue-specific PIN.

As the above results show, tissue-specific proteins usually function in conjunction with commonly expressed proteins. This implies that a balance in miRNA regulation might exist. Most simply, if a miRNA regulates some tissue-specific proteins, he should also include some function-related commonly expressed proteins in his regulatory perspective accordingly to ensure consistent regulation. Conversely, if a miRNA regulates a relatively high number of commonly expressed proteins which functions are shared by many tissue-specific biological processes, he is expected to affect more tissue-specific proteins' expression. Among the analyzed 294 miRNAs in our study, only 15 miRNAs regulate more commonly expressed proteins (n>20). Through counting the tissue-specific proteins regulated by them in all of the 10 tissues, we found that if a miRNA took a more enriched regulation on commonly expressed proteins, it would likely to also regulate expression of a relatively high number of tissue-specific proteins (n≥8) in more tissues (Figure 3A, Pearson correlation coefficient *r* = 0.7152, *p* = 0.0027).

Especially, 5 miRNAs (miR-1, miR-15a, miR-16, miR-21 and miR-155) regulated the largest number of commonly expressed proteins (n≥40), implying their broad and important roles. They are expressed in all of the 10 tissues [16]. To investigate the relationship between them and miRNA-regulated tissue-specific proteins in each tissue, we calculated the proportion of their targeted genes encoding tissue-specific proteins. Surprisingly, we found diverse tissue regulation modes executed by miRNAs (Figure 3B). For example, miR-15a regulated 12.7% of all miRNA-regulated tissue-specific proteins in the pancreas PIN, but did not affect any tissue-specific proteins in the skeletal muscle PIN. Just recently, the important role of miR-15a in regulating insulin synthesis has been disclosed [19], which is consistent with our analysis result. Another example is miR-1, a widely recognized muscle-specific miRNA [20]. Proportions of more than 15% were found in both the heart and skeletal muscle PINs, compared with lower proportions in the other tissue-specific PINs (Figure 3B).

## Discussion

In multicellular organisms, biological complexity not only can be reflected by numerous interactions between proteins, but also, more importantly, multidimensional regulations on them. These range from restricted tissue expression of proteins and precise subcellular localization, to ‘fine-tuning’ of protein production in posttranscriptional stage. As a vital node in posttranscriptional regulation, miRNAs maintain the dynamics of biological network by influencing protein output of target genes [7]. Notably, as a result of restricted tissue expression of proteins, only limited functional roles of miRNAs can be reserved in a particular cell or tissue. In the present study, we attempt to study the characteristics of tissue-specific gene regulation by miRNAs in the context of PIN (Figure 1A).

The fact of not all proteins being under miRNAs' monitoring implies selective regulation of miRNAs (Figure 2A). Universally expressed genes are more preferentially targeted by miRNAs than tissue-specific genes [21]. We further reveal that commonly expressed hub and super-hub proteins are generally under tightest control by miRNAs (Figure 2B). It can be expected that they are also strictly regulated at the transcriptional level [22]. On one hand, owing to sharing interactions with large number of partners, their inappropriate expression might lead to terrible cascade consequences; on the other hand, as they are located in the core of the network and participate in many biological processes, lack of control can cause coordination failure of related biological functions. ‘Fine-tuning’ of their protein abundance by miRNAs might contribute to reducing gene expression noise to maintain the stability of cellular environment [23]. In addition, we find that commonly expressed proteins would likely possess more mature regulatory mechanism of miRNAs than tissue-specific proteins (Figure 2B and C). Introducing the concept of degree density, we are able to reveal the close functional relationship between tissue-specific and commonly expressed proteins (Figure 1C). What does this mean for miRNA regulation? Our further finding suggests that miRNAs might attempt to find and construct a balance between their target genes encoding these two subunits of proteins. A positive correlation between the number of tissue-specific target genes and that of commonly expressed ones was found for the miRNAs with enriched regulation on core cellular components (Figure 3A). Five miRNAs regulate the largest number of commonly expressed proteins and correspondingly influence expression of more tissue-specific proteins (Pearson correlation coefficient *r* = 0.7152, *p* = 0.0027). But, surprisingly, we note that a particular miRNA may adapt diverse tissue regulation modes in different tissues (Figure 3B). Whether targeting more tissue-specific genes in a tissue means more important roles of a miRNA in the tissue? Regulating insulin synthesis, miR-15a provides a confirmatory example for this, which targets about 12.7% of the miRNA-regulated tissue-specific proteins in the PIN of pancreas [19]. Another example is miR-1, which targets 15.1% of the miRNA-regulated tissue-specific genes in heart, playing vital roles in cardiac electrophysiology and tissue remodeling [20], [24]. Taken together, our findings suggest miRNAs that interact with more commonly expressed genes can be expected to participate in important tissue-specific biological processes, despite such participation is also tissue-specific.

A considerable proportion of the analyzed miRNA-gene interactions here are predicted by ExprTarget, which integrates the current frequently used miRNA target prediction methods including miRanda, PicTar and TargetScan [9]. As genes with longer 3′ un-translated region (3′UTR) might have more predicted miRNAs regulating them, we evaluated the variation of different gene subunits in 3′UTR length. In the results, no significant variation in 3′UTR length was found in different gene subunits regardless of how genes were grouped, indicating that our analysis was not distorted by this (Table S2).

In conclusion, while false positive protein interactions and wrong miRNA targets may be inevitable, we still consider that integrating miRNA-gene interaction and protein interaction data facilitates better understanding tissue-restricted miRNA regulation. The continuously emerging vital roles of miRNAs raise the exciting possibilities that therapeutic manipulation of these pervasive regulators might benefit human disease. Our findings have implications not only for the miRNA related mechanism research but also for rationale screening of therapeutic and diagnostic miRNAs for various tissue diseases.

## Methods

### Tissue-specific PIN

We used the protein tissue expression data in HPRD of Release 9 [15]. Totally 10 human tissues were finally selected because of large quantity of experimentally validated proteins. They included brain, heart, kidney, liver, lung, skeletal muscle, pancreas, placenta, spleen and testis. Notably, the ubiquitously expressed proteins were also included in the protein list of each tissue. The Cytoscape [25] plug-in BisoGenet [14] was used to retrieve human experimentally validated protein interactions from 6 datasets (BIOGRID, INTACT, MINT, DIP, BIND and HPRD). After removing self-loops, isolated nodes and small network components, NetworkAnalyzer [26] calculated the degree value of each node representing protein which was then categorized following the classification scheme used by Lu et al. [27]. In addition, proteins represented as nodes were also sorted into four subunits according to their tissue expression specificity. In particular, proteins that expressed in all of the 10 tissues were defined as commonly expressed proteins, and proteins that expressed in only in less number of tissues (n≤3) were defined as tissue-specific proteins.

### MiRNA-gene interaction data

Firstly, we obtained the human miRNA expression profile of each of the above 10 tissues in mimiRNA [16]. Two databases miRSel [8] and ExprTargetDB [9] were then used together to search literature-reported or predicted target genes of each miRNA. In miRSel, four miRNA-gene interaction resources (Tarbase, miRecords, miR2Disease and miRSel) were all applied. In ExprTargetDB, only the prediction algorithm ExprTarget was selected with the threshed of exprscore set at 10. Finally, the target gene data of each miRNA was imported into the tissue-specific PINs if the miRNA was experimentally validated to be expressed in those corresponding tissues.

### Degree density

To evaluate how closely two classes of proteins or proteins belonging to the same class were interacted with each other, we introduced a concept of degree density here. Degree density quantitatively assesses the extent of sharing protein interactions between two classes of proteins as the maximum possibility of protein interactions is considered. The degree density for proteins belonging to the same class is calculated as the sum of actually occurred protein interactions subtracted by n·(n-1). The number n represents the sum of proteins. And, the degree density for proteins belonging to two different classes was calculated as the sum of actually occurred interactions between proteins from different classes subtracted by n·m. The numbers n and m represent the sum of proteins in the two classes, respectively.

### MiRNA with enriched regulation on commonly expressed proteins or tissue-specific proteins

We counted the sum of commonly expressed proteins regulated by the miRNAs expressed in each tissue. In total, 294 miRNAs were analyzed. Only 15 miRNAs that regulated >20 commonly expressed proteins were considered to take enriched regulation on commonly expressed proteins (Table S3). To investigate which miRNAs took enriched regulation on tissue-specific proteins in each tissue, we counted the sum of tissue-specific proteins regulated by miRNAs. Because less miRNA-gene interactions were found in tissue-specific subunit, we considered influencing ≥8 proteins as enriched regulation (Table S4).

### Statistical analysis

Data were analyzed using GraphPad Prism software (v5.0; GraphPad Software, Inc., USA). One-way analysis of variance (ANOVA) was used to compare difference among protein subunits and results were presented as mean ± SEM. The significant difference was determined as *p*<0.05. Pearson correlation coefficient was calculated to assess the correlation of the number of targeted commonly expressed genes of miRNAs (n>20) and the number of tissues in which a relatively high number of tissue-specific proteins regulated by miRNAs (n≥8) could be found.

## Supporting Information

Table S1 Comparison of co-expression of different tissue-specific gene targets with the corresponding miRNAs.
(DOC)

Table S2 Comparison of different gene target subunits in the 3′UTR length.
(DOC)

Table S3 MiRNAs with enriched regulation on commonly expressed proteins.
(DOC)

Table S4 MiRNAs with enriched regulation on tissue-speific proteins.
(XLS)
