# Correction: MicroRNA Regulation and Tissue-Specific Protein Interaction Network

**DOI:** 10.1371/annotation/60addd69-bd2d-4ecf-8369-6a5a1bf2cd04

**Published:** 2011-10-21

**Authors:** Wenliang Zhu, Lei Yang, Zhimin Du

The authors have the following funding information to report: "This work was supported by the National Natural Science Foundation of China (81072639). The funders had no role in study design, data collection and analysis,decision to publish, or preparation of the manuscript." 

