# Supplementary material for: MicroRNA Regulation and Tissue-Specific Protein Interaction Network
Source: PLoS One. 2011 Sep 27;6(9):e25394. doi: 10.1371/journal.pone.0025394 (PMC3181334; doi:10.1371/journal.pone.0025394)
Supplement: Table S1 — Comparison of co-expression of different tissue-specific gene targets with the corresponding miRNAs. (DOC) [file pone.0025394.s001.doc]

**Table S1**. Comparison of co-expression of different tissue-specific gene targets with the corresponding miRNAs

| Number of tissues | Percentage of miRNA-gene interactions (Average ± SD, %) |
| --- | --- |
| 1-3 | 91.1 ± 6.1 |
| 4-6 | 91.5 ± 6.0 |
| 7-9 | 91.0 ± 6.0 |
| 10 | 91.7 ± 5.9 |

Comparison of co-expression of different tissue-specific gene targets with the corresponding miRNAs. Percentage of miRNA-gene interactions: the number of acturally happened miRNA-gene interactions divided by the total number of miRNA-gene interactions to a given subunit of gene targets in a tissue-specific PIN. There were no statistical significant difference between percentage of miRNA-gene interactions means at *p* = 0.05.
