# Supplementary material for: MicroRNA Regulation and Tissue-Specific Protein Interaction Network
Source: PLoS One. 2011 Sep 27;6(9):e25394. doi: 10.1371/journal.pone.0025394 (PMC3181334; doi:10.1371/journal.pone.0025394)
Supplement: Table S2 — Comparison of different gene target subunits in the 3′UTR length. (DOC) [file pone.0025394.s002.doc]

**Table S2**. Comparison of different gene target subunits in the 3'UTR length

| Tissue | P-value/Adjusted (tissue expression specificity) | P-value/Adjusted (Degree) |
| --- | --- | --- |
| brain | 0.2481/0.6180 | 0.2582/0.9469 |
| heart | 0.6180/0.6180 | 0.4097/0.9469 |
| kidney | 0.4336/0.6180 | 0.9469/0.9469 |
| liver | 0.0461/0.6180 | 0.4246/0.9469 |
| lung | 0.0283/0.2547 | 0.8964/0.9469 |
| skeletal muscle | 0.1698/0.6180 | 0.3643/0.9469 |
| pancreas | 0.0473/0.3416 | 0.7870/0.9469 |
| placenta | 0.0188/0.1880 | 0.8465/0.9469 |
| spleen | 0.0488/0.3416 | 0.9132/0.9469 |
| testis | 0.2146/0.6180 | 0.6378/0.9469 |

Comparison of different gene target subunits in the 3'UTR length. The miRNA gene targets were classified into different subunits according to their tissue expression specificity (number of tissues: 1-3, 4-6. 7-9, 10) or degree (Peripheral-A, Peripheral-B, Hub&Super-hub). The 3'UTR length of gene was retrieved from the TargetScan website (<http://www.targetscan.org/>). The calculation of P-values was based on One-way ANOVA test. The *P*-values were then adjusted for multiple comparisons using the Benjamini and Hochberg method. There were no statistical significant difference between 3'UTR length means at adjusted *P*-value = 0.05 regardless of how genes were grouped.
