# Supplementary material for: MicroRNA Regulation and Tissue-Specific Protein Interaction Network
Source: PLoS One. 2011 Sep 27;6(9):e25394. doi: 10.1371/journal.pone.0025394 (PMC3181334; doi:10.1371/journal.pone.0025394)
Supplement: Table S3 — MiRNAs with enriched regulation on commonly expressed proteins. (DOC) [file pone.0025394.s003.doc]

**Table S3**. MiRNAs with enriched regulation on commonly expressed proteins

| miRNA | the number of regulated proteins that are commonly expressed (n) |
| --- | --- |
| miR-155 | 48 |
| miR-16 | 44 |
| miR-21 | 42 |
| miR-15a | 41 |
| miR-1 | 40 |
| miR-9 | 33 |
| miR-200b | 27 |
| miR-145 | 26 |
| miR-200c | 26 |
| miR-206 | 26 |
| miR-125b | 24 |
| miR-30b | 23 |
| let-7b | 21 |
| miR-146a | 21 |
| miR-221 | 21 |
